# EXAMINING DIFFERENCES IN SLEEP QUALITY AMONG CAREGIVERS FOR PERSONS LIVING WITH AND WITHOUT DEMENTIA

**DOI:** 10.1093/geroni/igad104.0504

**Published:** 2023-12-21

**Authors:** Darina Petrovsky, Sophia Geisser, Elizabeth Luth

**Affiliations:** Rutgers University, Jackson, New Jersey, United States; The University of Alabama, Tuscaloosa, Alabama, United States; Rutgers University, New Brunswick, New Jersey, United States

## Abstract

Sleep disturbances among caregivers are linked to increased burden and worse mental health outcomes. Caregivers for persons living with dementia experience more sleep disturbances than non-caregivers. Few studies examine whether sleep quality differs between dementia and non-dementia caregivers. This study analyzes whether dementia caregivers experience worse sleep quality than non-dementia caregivers. Using 2017 National Study of Caregiving, National Health and Aging Trends Study, and time diary interview data, we examined the relationship between caregiver sleep quality (“excellent/very good” versus “good/fair/poor” the previous night) and care recipient’s dementia status (“probable” versus “no”) for 1,374 caregivers in a series of nested multi-variable logistic regression models, controlling for the caregiver and care recipient sociodemographic characteristics, relationship quality, and other controls. Caregivers’ mean age was 62.3 years old (SD=14) and about two-thirds were women (68%), non-Hispanic White (69.4%), and married (66%) Thirty-eight percent cared for persons living with dementia. In the bivariate model, being a caregiver for someone living with dementia was associated with significantly lower odds of reporting “excellent/very good” sleep quality (OR: 0.78, 95%CI: 0.62-0.97, p=0.02). This relationship persisted independent of caregiver and care recipient demographic characteristics. However, caregiver perceptions of the quality of their relationship with the care recipient and the benefits of their caregiving role explained the relationship between care recipient dementia status and caregiver sleep quality ratings (AOR: 0.80, 95%CI: 0.63-1.02, p=0.08). Addressing caregiver burden and relationship quality may help improve sleep quality among dementia caregivers and reduce disparities in sleep quality between dementia and non-dementia caregivers.

